# Pyrosequencing as a method for SNP identification in the rhesus macaque (*Macaca mulatta*)

**DOI:** 10.1186/1471-2164-9-256

**Published:** 2008-05-29

**Authors:** Jessica A Satkoski, RS Malhi, S Kanthaswamy, RY Tito, VS Malladi, DG Smith

**Affiliations:** 1 Department of Anthropology, University of California-Davis, One Shields Avenue, Davis, CA, USA; 2 Department of Anthropology, University of Illinois-Urbana-Champaign, Matthews Avenue, Urbana, Illinois, USA; 3 Institute for Genomic Biology, University of Illinois-Urbana-Champaign, West Gregory Drive, Urbana, Illinois, USA; 4 California National Primate Research Center, University of California-Davis, Hutchison Road, Davis, CA, USA

## Abstract

**Background:**

Rhesus macaques (*Macaca mulatta*) are the primate most used for biomedical research, but phenotypic differences between Indian-origin and Chinese rhesus macaques have encouraged genetic methods for identifying genetic differences between these two populations. The completion of the rhesus genome has led to the identification of many single nucleotide polymorphisms (SNPs) in this species. These single nucleotide polymorphisms have many advantages over the short tandem repeat (STR) loci currently used to assay genetic variation. However, the number of currently identified polymorphisms is too small for whole genome analysis or studies of quantitative trait loci. To that end, we tested a combination of methods to identify large numbers of high-confidence SNPs, and screen those with high minor allele frequencies (MAF).

**Results:**

By testing our previously reported single nucleotide polymorphisms, we identified a subset of high-confidence, high-MAF polymorphisms. Resequencing revealed a large number of regionally specific SNPs not identified through a single pyrosequencing run. By resequencing a pooled sample of four individuals, we reliably identified loci with a MAF of at least 12.5%. Finally, we found that when applied to a larger, geographically variable sample of rhesus, a large proportion of our loci were variable in both populations, and very few loci were ancestry informative. Despite this fact, the SNP loci were more effective at discriminating Indian and Chinese rhesus than STR loci.

**Conclusion:**

Pyrosequencing and pooled resequencing are viable methods for the identification of high-MAF SNP loci in rhesus macaques. These SNP loci are appropriate for screening both the inter- and intra-population genetic variation.

## Background

Rhesus macaques (*Macaca mulatta*) are used more extensively as animal models for the study of human disease than any other primate species. They provide the primary model for research in infectious diseases, reproductive biology, behavior, neuroscience and immunology. More recently, they have been employed in research and vaccine development for the human immunodeficiency virus (HIV) [1-3]. The severe shortage of rhesus macaques as subjects for biomedical research prompted the establishment of national centers for breeding them in the US [4-6]. After their exportation from India ceased in 1978, China became the principal supplier of rhesus macaques to these centers and, thus, domestically bred rhesus macaques represent both countries of origin with only negligible contributions from rhesus macaques from other countries [7]. The particular shortage of Indian-derived rhesus macaques available for use as subjects in biomedical research and their desirability over Chinese rhesus macaques have led to efforts to acquire Indian-like rhesus macaques from sources outside India, such as Nepal and Bangladesh [7], and to establish close relationships with the National Center for Primate Breeding and Research currently under construction in Bombay, India.

Nozawa et al. [8] were the first to characterize genetic polymorphisms in a broad range of Asian species of genus Macaca, including *M. mulatta*. They reported differences in allele frequencies for electrophoretically defined protein polymorphisms among regional populations of the species. Later, short tandem repeat (STR) polymorphisms were identified in rhesus macaques by using the polymerase chain reaction (PCR) and cross-amplification with human primers [9-11]. Variation in the frequencies of MHC alleles [12-16], mitochondrial DNA (mtDNA) variation, restriction-site polymorphism (RFLP) haplotypes [17,18] and sequence [19,20], and Y-chromosome haplotypes [21] were also characterized.

Since then, specific genetic differences between populations of rhesus macaques from China and India have been characterized based on electrophoretically defined protein polymorphisms [22,23], STR polymorphisms [24-29], major histocompatibility (MHC) alleles [30-32], mtDNA [17,28,33] and, most recently, single nucleotide polymorphism (SNPs) [34-37]. Unfortunately, the number of polymorphisms represented above is far too small, and their distribution throughout the genome too broad, to permit whole genome studies of quantitative trait loci (QTL) and disease association [35].

Until recently, STR loci, numbering in the tens of thousands in most mammalian genomes, have been the preferred polymorphisms for characterizing genetic variability and estimating parameters useful for genetic management. STRs serve this purpose because they are neutral to selection pressure, exhibit multiple alleles (sometimes dozens), and hence demonstrate high levels of heterozygosity under equilibrium conditions. They are also spread throughout the genome making it possible to use them create a linkage map the rhesus genome [38], albeit at a density insufficient for whole genome association studies.

The recent completion of the rhesus genome map [39], however, has made the discovery of SNPs much easier [35-37]. While SNPs are typically biallelic and, therefore, exhibit fewer alleles and lower expected heterozygosity (gene diversity) than many STRs, they have the following advantages over STRs, making them more desirable for genetic management and for biomedical and/or genomic research. First, several hundred STRs would be required to achieve reliable estimates of the phylogenetic relationship and divergence time between Indian and Chinese rhesus macaques [40], a process would be both time-consuming and expensive. In contrast, several thousand SNPs can be simultaneously assayed through automation, reducing both the size of the confidence intervals around parameter estimates and cost. Second, SNPs are free of many of the sources of error (e.g., non-specific amplification, shadow bands, null alleles) that characterize STR genotypes. Being biallelic, the assignment of SNP alleles is free from the subjectivity that plagues STR genotyping. Additionally, SNPs can be more reliably genotyped with identical results in different laboratories [41].

Additionally, when carefully selected, SNPs can be expected to exhibit less homoplasy than STRs, and hence provide fewer false signals of common ancestry (thus, SNPs will be more reliable and informative as ancestry informative markers, or AIMs). Because STRs evolve following a stepwise mutation model and exhibit size constraints on mutation rates and direction, genetic signatures resulting from genetic bottlenecks (or expansions) are more ambiguous than for SNPs, which follow an infinite allele model of mutation [42]. Moreover, size constraints on the evolution of STRs, absent in SNPs, can lead to serious mis-specification of branch lengths and topology of phylogenetic trees [43].

STRs have been used successfully to identify linkage disequilibrium between pairs of loci, such as CD4 [44] and G6PD [45]. However, SNPs are more plentiful in the genome than STRs by a half dozen orders of magnitude and can be located quite close to a greater number of functional genes, allowing the creation of a high-density map throughout the genome. SNPs located at various distances from the target of selection exhibit strong linkage disequilibrium and exhibit values of Fst that systematically decline with distance from the target of directional selection, a pattern that has been observed in the human Duffy blood group [46], lactase [47] loci, and loci that influence human skin pigmentation [48,49].

SNPs with high levels of heterozygosity (e.g., between 0.4 and 0.5) will provide estimates of parameters important for genetic management (e.g., gene diversity, genetic subdivision and average inbreeding coefficient). The extraordinary quantity of SNPs present in most mammalian genomes insures that the large number of highly informative SNPs necessary for this purpose can be found.

A large number of SNPs evenly spaced throughout the genome (the current STR map includes only 241 loci [38]), could be used in many types of genomic research, including creating a haplotype map of the rhesus macaque genome and searching for evidence of selection, studying the histories/phylogenies of multiple genes linked to SNPs (SNPsters- [50]), mapping of QTLs or other important phenotypes [see [51,52], and [53] for examples using the baboon genome]. In addition, a comparison of the human and chimpanzee genomes has detected genes with unusually high ratios of non-synonymous to synonymous mutations, suggesting selection on genes influencing immune defense and tumor suppression in the human lineage [56]. However, this inference requires the assumption that the chimpanzee always expresses the ancestral form of these genes, an assumption that is not always valid. The availability of a rhesus SNP map would allow testing of this assumption. Thus, a detailed knowledge of population structure of rhesus macaques based on SNPs at various distances and on different chromosomes is crucial to the interpretation of studies of association between specific loci and disease etiology and susceptibility. The pyrosequencing strategy could provide these SNPs relatively cheaply and quickly. We subjected a DNA sample from a Chinese rhesus macaque to pyrosequencing. After parsing the resulting pyrofragments, we compared them to the newly completed rhesus genome to identify candidate SNPs. We selected a sample of these SNPs and conducted the following three tests to evaluate pyrofragment comparison as a method of SNP detection in the rhesus macaque.

### Test 1

Fragment Resequencing. Many of the pyrosequenced fragments overlapped each other, ranging from zero to three overlaps (fragments with more than three overlaps were removed from the data set, per [37]). The quality of each base in the sequence was assessed with the program Phred [54,55]. Phred score is assigned relative to the probability of an incorrect base call, with each 10-point increase equating to a 10-fold decrease in the probability of a miscalled base. (For example, a score of 20 equals 99% base call accuracy, while a score of 30 indicates 99.9% accuracy.) The Phred scores in these fragments ranged from one to 33. We resequenced a sample of these pyrofragments in both the original pyrosequenced individual and several other rhesus macaques, to evaluate the impact of overlap number and Phred score on the accuracy of our SNP detection algorithm. The goal of this test was determine an appropriate cutoff for overlap number and Phred score during SNP locus selection.

### Test 2

Screening for Informative SNPs. Because all SNPs were detected through the comparison of a pyrofragments from a single Chinese rhesus to the genome of a single Indian rhesus, we resequenced a pooled DNA sample of four different, geographically diverse rhesus macaques. By this method, only SNPs located on at least one of the 4 orthologous chromosomes in the pool would be verified (MAF of at least 12.5%), suggesting that the heterozygosity under equilibrium conditions exceeds 0.22. Resequencing a pooled DNA sample will allow us to screen a large group of loci for those most likely to be informative in a larger population of animals.

### Test 3

Detecting Geographic Variation. Given the large number SNPs identified from a single pyrosequencing run (~23,000), it is necessary to develop a method to test the efficacy with which they measure genomic variation. The goal of this test was twofold. First, we sought to not just identify SNP loci with a MAF of greater than 12.5%, but to identify markers meeting this criterion that were also AIMs. Second, we wanted to test the utility of randomly identified SNP loci for population genetic analysis compared with STR loci. To this end, 96 SNPs fitting the criteria established by the first two tests were chosen for genotyping by the Illumina GoldenGate Assay System in a sample of 95 geographically diverse Indian and Chinese rhesus macaques. These same 95 DNA samples were amplified at 23 microsatellite loci dispersed throughout the genome, and the utility of these markers for capturing the genetic variation present in the populations were compared.

## Results

### SNP Detection

Details of our SNP detection protocol can be found in [37], and the bioinformatic methods are described in [57]. Our SNP database can be searched by chromosome, base pair location, or unique pyrofragment ID at our website, located at .

### Fragment Resequencing

**Figure 1:**
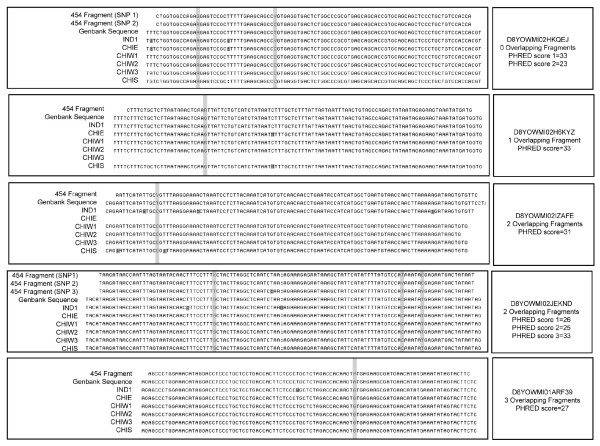
**Sanger re-sequencing of the 454 pyrosequenced DNA fragments**. Although few of the identified SNPs are geographically informative, many of the resequenced individuals contain unique SNPs (marked in dark grey).

Individual and Primer information are shown in Table 1. There were no differences between the pyrosequenced 454 fragments and the Sanger re-sequenced CHIW sample (generated from the same individual), supporting the low pyrosequencing error rate suggested by the Phred scores, as shown in Figure 1. The only exception is in fragment D8YOWMI02H6KYZ, where Sanger sequencing revealed individual Sch00R1684 to be a heterozygote. A minimum Phred score of 20 (99% accuracy) was considered an acceptable error rate for all subsequent tests. To increase our confidence that identified SNPs were true polymorphisms rather than sequencing errors, a SNP had to have been identified in a minimum of two pyrofragments to be considered for further analysis. These sequence comparisons demonstrate, though, that the scores assigned by the Phred program are accurate, and can be used as a general guideline to allow future SNP selection without having to verify the polymorphism through sequencing.

**Table 1:** Sequence and position information for primers used to replicate 454 pyrosequenced fragments.

**Pyrofragment ID**	**Chromosome**	**Position**	**Change**	**Overlapping Fragments**	**PHRED**
D8YOWMI02HKQEJ	1	732396	A->G	0	33
D8YOWMI02HKQEJ	1	732419	T->C	0	23
Forward Primer:	5'TTCAACTTGCTCCAGTAGGC3'				
Reverse Primer:	5'TGCTCTGATTTCAGGAGTTCAA3'				
D8YOWMI02IZAFE	20	73952158	A->G	2	31
Forward Primer:	5'GAGATGGTGTTAGCCGCTTT3'				
Reverse Primer:	5'TTCAAGATTCTCCACACTCATGTT3'				
D8YOWMI02H6KYZ	13	77238559	C->T	1	33
Forward Primer:	5'AAACTTCCGTTTGATCACAAGTC3'				
Reverse Primer:	5'CCTCAGTGGATTTCAACAACAA3'				
D8YOWMI02JEKND	14	101674555	A->G	2	26
D8YOWMI02JEKND	14	101674561	G->A	2	25
D8YOWMI02JEKND	14	101674616	C->A	2	30
Forward Primer:	5'GCTTACATCTTTTTAGTAACCAGCA3'				
Reverse Primer:	5'CGGGGTTTTCGTAAGAGAGA3'				
D8YOWMI01ARF39	2	37080526	T->C	3	27
Forward Primer:	5'GGTCTTTTAATTCTGTATCTGGATGTC3'				
Reverse Primer:	5'GAATCCCTTATCATCTCAGCAA3'				

Also shown in Figure 1, the SNPs identified through comparison of the 454 fragment with the NCBI rhesus genome do not tend to be restricted to a particular regional sample; indeed, although the NCBI genome was created from an Indian individual, the Indian rhesus included in our regional sample always carried the same allele as the pyrosequenced (Chinese) individual, suggesting that a very low percentage of the identified SNPs qualify as AIMs.

However, a significant result of our test was that the comparison of regional samples revealed a great deal of previously undiscovered sequence polymorphism, indicated by dark grey boxes in Figure 1. This polymorphism suggests that genomic comparison of individuals from different geographic regions has the potential to quickly generate many more informative SNP loci. This result suggests that pyrosequencing additional, regionally variable, individuals has the potential to reveal much more of the variation present in the rhesus genome.

### Screening for Informative SNPs

48 SNPs were selected to screen the pooled DNA sample (individuals listed in Table 2). At least two SNPs were chosen on each chromosome, one on each arm. Primers were successfully generated for 43 loci. Of these 43, 60% (N = 26) were confirmed as polymorphic. The remaining 40% that could not be verified are classified as low-frequency SNPs, and in fact may have been alleles private to the original pyrosequenced individual. Consistent with the results of the first test, between one and 13 additional SNPs were identified in each of the 43 amplicons. All four individuals, when sequenced individually at three randomly selected loci, produced amplicons for two of the loci, while only three individuals amplified at a third. The former loci all exhibited minor allele frequencies of 25%, which the latter exhibited a minor allele frequency of 33%. Thus the strategy of using a pooled sample is an effective way to select loci with minor allele frequencies of at least 12.5%. Of course, use of sample pools representing only 2 or 3 samples should identify SNPs with proportionately higher MAFs.

**Table 2:** Provenience and accession information for the individuals included in all experiments described above.

**Test**	**Sample**	**Origin**	**Source^a^**	**Haplogroup**	**Accn. #**
Pyrofragment Resequencing	CayAD41	Uttar Pradesh, India	CPRC	IND1	AY647044
	Suz34497	Jiangsu, China	CNPRC	CHIE	AY646935
	Sch00R1684	Sichuan, China	VBS, TSS	CHIW1	DQ373245
	Sch00706	Sichuan, China	VBS, TSS	CHIW2	DQ373240
	Sch01R1119	Sichuan, China	VBS, TSS	CHIW3	DQ373263
	Gud963452	Guangdong, China	VBS	CHIS	DQ373092

Screening for Informative SNPs	Sch01R0670	Sichuan, China	VBS, TSS	CHIW3	DQ373257
	Viet24460	Vietnam	CNPRC	CHIS	AY464965
	Bur46	Myanmar	BPRC	BURM	N/a
	Cay51L	Uttar Pradesh, India	CPRC	IND1	AY647028

^a^CPRC, Caribbean Primate Research Center; CNPRC, California National Primate Research Center; VBS, Valley Biosystems; TSS, Three Springs Scientific, Inc.; BPRC, Biomedical Primate Research Center, Rijswijk, The Netherlands.

### Detecting Geographic Variation

Of the 95 individuals shown in Table 3, 79 produced viable genotypes for at least 95% of the 96 loci (listed in Table 4). Ninety-two of the loci submitted for genotyping produced analyzable data. Fourteen of these loci were monomorphic in all individuals, suggesting that they were either extremely low-frequency SNPs, or a mutation novel to the pyrosequenced animal.

**Table 3:** Samples submitted for SNP genotyping.

**Country of Origin**	**mtDNA Haplotype^a^**	**Geographic Origin**	**Sample Size**	**Source^b^**
China (N = 43)	CHIE	Guangdong	9	VBS
		Jiangsu	10	CNPRC
	CHIW	Sichuan (CHIW1)	9	VBS, TSS
		Sichuan (CHIW2)	8	VBS, TSS
		Sichuan (CHIW3)	4	VBS, TSS
	CHIS	Guangdong	3	VBS

India (N = 36)	IND1	Uttar Pradesh	24	CPRC
		Delhi	4	WNPRC
		Kashmir	3	UM
	IND2	Unknown	3	ONPRC
		Unknown	1	CNPRC
		Delhi	1	WNPRC

^a^GenBank accession numbers available from authors upon request.

^b^VBS, Valley Biosystems; CNPRC, California National Primate Research Center; TSS, Three Springs Scientific, Inc.; CPRC, Caribbean Primate Research Center; WNPRC, Wisconsin National Primate Research Center, UM, University of Miami; ONPRC, Oregon National Primate Research Center.

**Table 4:** The 96 SNP loci submitted for genotyping.

**Chromosome**	**No. Markers**	**Adjacent Marker Distance (Range in Mb)**	**Total Marker Distance (Mb)**
1	3	42.0 – 54.0	96.1
2	5	1.1 – 65.2	91.8
3	5	2.8 – 86.7	110.9
4	5	3.8 – 62.3	142.4
5	6	0.1 – 15.2	24.5
6	6	1.5 – 91.3	114.1
7	5	1.7 – 15.1	29.9
8	5	1.2 – 77.8	102.7
9	5	0.2 – 46.9	80.4
10	5	4.0 – 26.5	55.1
11	6	0.2 – 62.9	90.7
12	3	6.0 – 29.1	35.1
13	5	0.1 – 21.2	37.8
14	6	2.4 – 54.2	90.8
15	5	0.5 – 25.7	85.5
16	3	5.4 – 13.2	18.5
17	4	2.4 – 18.1	24.7
18	4	6.4 – 21.9	35.9
19	1	-	-
20	5	0.03 – 54.2	75.0
X	4	0.3 – 39.5	56.1

Adjacent marker distance is the smallest and largest number of megabases between two adjacent markers, while total marker distance indicates the total number of megabases spanned by all the markers.

**Figure 2:**
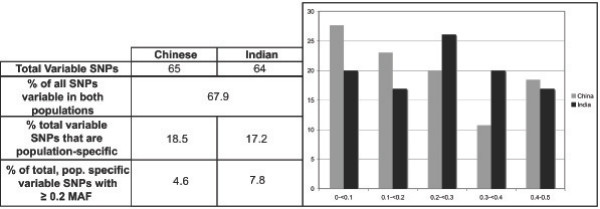
**Number of SNPs in each MAF class**. Expressed as a percent of the total for that sample, to account for differing numbers of variable SNPs in each population.

Sixty-five SNPs were variable in Chinese animals, and sixty-four were variable in Indian animals (one locus did not amplify in any Indian individuals). Of all the variable SNPs, 67.9% were polymorphic in both Chinese and Indian individuals. A very small proportion of the polymorphisms were unique to either population – only 18.5% and 17.2% of markers were ancestry informative in Chinese and Indian populations, respectively. Of these private polymorphisms, the distribution of MAF is shown in Figure 2. There is no significant difference between the MAF of the Chinese or Indian samples for each frequency class (2 sample t-test, *p *= 1), nor is there a significant relationship between the MAF class and the number of SNPs. The average heterozygosity in the Chinese sample was 0.25 ± 0.18, while in the Indian sample, it was slightly higher, at 0.28 ± 0.18. We were not able to detect any significant difference in linkage disequilibrium between the Chinese and Indian individuals (data not shown).

**Figure 3:**
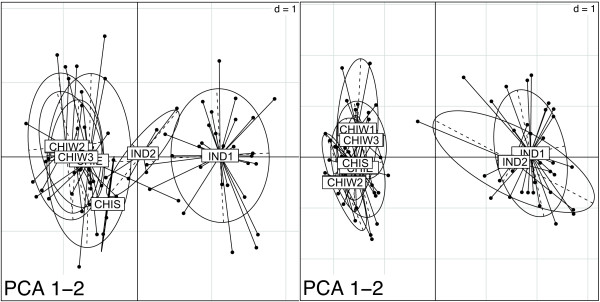
**Principle Component Analysis**. A comparison of principle components 1 and 2 for 80 individuals assayed at 23 STR loci (left) and 92 SNP loci (right).

The results of the STR analysis are shown in Table 5. The Chinese sample demonstrated unique alleles for each of the 23 loci, with an average of 4.7 unique alleles per locus. The Indian sample only demonstrated unique alleles at 7 loci, with an average of 1.6 unique alleles per locus. The observed heterozygosities of the Chinese and Indian sample were 0.76 ± 0.11 and 0.68 ± 0.14, respectively. The results of the principal component analysis (PCA) for the SNP and STR data are shown in Figure 3.

**Table 5:** Summary information for the 23 STR loci included in the study.

		**China**			**India**		
		
**Locus**	**Rhesus Chrom**.	**No. Alleles**	**Obs. H**	**Exp. H**	**No**. **Alleles**	**Obs. H**	**Exp. H**
D1s548	1	7	0.74	0.73	8	0.61	0.69
D3s1768	2	15	0.84	0.90	17	0.77	0.82
D7s1826	3	8	0.74	0.77	6	0.71	0.71
D7s794	3	7	0.58	0.66	6	0.73	0.68
D6s501	4	8	0.70	0.73	8	0.81	0.75
D4s1626	5	20	0.62	0.93	22	0.65	0.91
D5s1457	6	10	0.62	0.77	9	0.71	0.64
270o7	6	6	0.68	0.63	6	0.54	0.60
D15s644	7	19	0.63	0.89	18	0.75	0.85
D8s1466	8	12	0.77	0.86	11	0.52	0.62
D8s1106	8	13	0.76	0.86	12	0.79	0.85
D10s1432	9	8	0.71	0.77	6	0.5	0.65
271j8	9	11	0.80	0.83	6	0.95	0.81
AGAT007	11	13	0.76	0.88	14	0.93	0.88
AGAT001	12	9	0.86	0.84	8	0.73	0.76
D11s2002	14	8	0.64	0.79	7	0.62	0.73
D9s921	15	8	0.79	0.78	8	0.47	0.57
D9s934	15	9	0.72	0.75	9	0.79	0.81
270e8	16	9	0.74	0.80	9	0.86	0.77
D13s765	17	18	0.93	0.91	9	0.48	0.74
D13s318	17	18	0.95	0.91	14	0.48	0.75
D18s537	18	5	0.51	0.36	5	0.65	0.64
D18s861	18	3	0.51	0.51	4	0.70	0.65
Mean	-	10.6 ± 4.7	0.76 ± 0.11	0.81 ± 0.08	9.7 ± 4.6	0.68 ± 0.14	0.73 ± 0.09

## Discussion

The results shown in Figure 2 suggest that the allelic variants discovered through comparison of the Chinese 454 fragments and the Indian NCBI rhesus genome are probably broadly polymorphic in the species, rather than being confined to a particular region. These results conflict with those reported by Ferguson et al. [36] for SNPs identified in 3' regions of coding sequences of the rhesus genome, and by Hernandez et al. [35] for SNPs identified by sequencing several ENCODE regions. While the SNPs identified by Ferguson et al. [36] and Hernandez et al. [35] will be extremely useful as AIMs for differentiating between rhesus macaques originating in India and China and for estimating the level of admixture in hybrid rhesus macaques with ancestry from both regions, they might be unsuitable for studies of the structure of the rhesus macaque genome if they prove to be unrepresentative of this structure.

Additionally, our comparison of regional samples revealed a great deal of previously undiscovered sequence polymorphism, indicated by dark grey boxes in Figure 1. This polymorphism suggests that genomic comparison of individuals from different geographic regions has the potential to quickly generate more informative SNP loci in the process of verifying SNPs that are discovered in the future using the methodology we have used. This might be of particular importance for identifying derived SNPs in non-rhesus species, such as *M. fascicularis*.

A single pyrosequencing run cannot discover all the variation present in the rhesus macaque genome. Because a single run does not produce complete coverage of the genome. However, as shown in Figure 1, the pyrosequencing of additional regional samples will reveal more of these region-specific polymorphisms. Thus, we can expect that many more additional candidate SNPs than the 23,000 we have already identified will be revealed using this re-sequencing procedure, when both additional animals and regionally variable animals are pyrosequenced. Moreover, recent advances in 454 technologies have significantly increased the efficiency of the pyrosequencing reaction, nearly tripling the number of reads per run and increasing by two and one-half fold (to 250 bp) the size of each read. Thus, we might be able to detect approximately 60,000 SNPs (two and one-half fold larger than the number identified in fragments of average size 103 bp) from each additional pyrosequencing experiment. Recently, researchers have developed a method for discovering SNP loci and estimating MAF in a single run, by pooling individuals and digesting with *Hae*III, selecting fragments in the 70–200 bp range (creating a reduced representation library, or RRL) and resequencing. This method has been used to successfully identify large numbers of SNPs in domestic cattle [58] and could easily be adapted to the rhesus macaque.

Finally, the SNP data presented in this study address many of the concerns of researchers regarding STR data. The SNPs in this study were identified from a pool of ~23,000 candidates. As shown in Figure 1, the error rate of the Illumina SNP genotyping process is very low, and PHRED score appears to be quite useful as a measure of genotype confidence. This is quite different from STR genotyping, where genotype reliability is generally a measure of how many times it can be replicated [59]. Additionally, because the SNP loci are biallelic, there is no subjectivity necessary in the assignment of genotypes, nor is there a need for a "universal" allelic ladder, such as that used for human forensic STR testing [60-62].

In addition to replicability, SNP genotypes can be collected more quickly than STRs. The SNP data presented in this paper was collected in a single run, while the comparable STR data required hundreds of separate PCR reactions and at least four "pool-plexed" passes or two multiplex passes through the sequencer to produce complete, high-confidence genotypes.

However, efficiency and thrift are poor tradeoffs if the resultant data does not adequately assay inter-and intra-population genetic variation. Even with the relatively low number of either Chinese- or Indian-specific polymorphisms, as shown in Figure 3, our loci, *when taken together*, provide sufficient phylogenetic signal to discriminate Indian and Chinese rhesus populations. The separation of the IND1 and Chinese populations using STR loci alone is not nearly as complete, although the position of IND2 individuals in the STR-based PCA in Figure 3 could be due to underlying genetic structure not detected by the SNPs. This PCA, in combination with the heterozygosity measures from both types of loci, suggest not that the STR loci currently in use are biased [28], but that that genetic data collected from SNP loci simply provide a more complete estimate of the genetic variation in the rhesus genome. There was at least one SNP on each arm of each chromosome (excluding chromosome 19), while the distribution of the STRs was not as extensive. Nearly 70% of our SNP loci were variable in both Chinese and Indian individuals, making them useful for comparing the breadth of genomic variation between these populations, something that cannot be done with AIMs. Within populations, the average observed heterozygosity of our SNP loci was generally lower than the comparable STR data. However, in both regional samples, high and low MAF loci were equally common. If a high MAF is the primary criterion for inclusion in a SNP-based genetic management panel, pyrosequencing appears to be an especially useful method for quickly identifying large numbers of these loci. Post-identification, pooled resequencing as described above identifies loci with a MAF of at least 12.5% with a high degree of accuracy (although this percentage depends on the number of samples included in the pool).

## Conclusion

In this study, we sought to expand on the SNP detection methods presented by Malhi et al. [37], by applying three tests to the approximately 23,000 previously identified SNPs. First, we replicated several of the pyrosequenced fragments with varying Phred scores, to both check for sequencing error and to screen for additional, regionally specific polymorphisms. From this, we found that the Phred score was an accurate representation of sequencing error rate, and set a minimum Phred score of 20, or 99% base call accuracy, for all subsequent tests. Second, we used a pooled DNA sample containing four different individuals to resequence 48 SNP loci. By using this technique to screen for polymorphic loci, and then sequencing animals individually to quantify the allele frequencies, we were able to screen for loci with MAF of at least 12.5. Third, we genotyped loci in a larger sample of 95 regionally variable macaques. We found that although a small percentage of the loci qualified as AIMs for this sample, a large proportion of loci had MAF in the 40–50% range, suggesting that our method of SNP identification is appropriate when the goal is the assessment of within-population genetic variation. Finally, we were able to demonstrate that the SNP loci were at least as useful as STRs for screening within-population genetic variation, and were better at between-population discrimination for the IND1 and Chinese individuals.

## Methods

### SNP Detection

A DNA sample from a rhesus macaque from Sichuan Province, China, with the ChiW1 mtDNA haplogroup was submitted to 454 Life Sciences™ for pyrosequencing. This sample was chosen because there is a high probability, based on its mtDNA haplogroup [e.g., see [33]] and its genotypes for five ancestry informative STR loci [e.g., see [27,28]], that this animal has no admixture with eastern Chinese rhesus macaques in its ancestry. We developed bioinformatic tools to align these pyrofragments with the Baylor Genome Center/Washington University Genome Center's Indian rhesus genome sequence [39,63] and to output these alignments.

### Fragment Resequencing

Eight of the approximately 23,000 candidate SNPs [37] were selected for Sanger sequencing, representing SNPs identified in a varying number of overlapping fragments (0–3 fragments) and a range of PHRED scores [23-33]. After choosing these loci, 150 bp of flanking sequence was retrieved from Build 1.1 of the NCBI rhesus macaque genome. When the 454 fragments contained more than one SNP (e.g. fragments D8YOWMI02HKQEJ and D8YOWMI02JEKND), the NCBI sequence length was calculated so that all the SNP loci were represented. These sequences were entered into the Primer3 web interface [64] and oligonucleotide primers were designed to amplify an approximately 200 bp fragment containing the SNP. The validity of these primers, listed in Table 1, was tested using the program Amplify 3.1 for Macintosh.

Six *Macaca mulatta *individuals were selected for SNP confirmation. DNA was extracted from EDTA-preserved blood samples using the QIAmp Blood Mini Kit (Qiagen, Valencia CA). All of these individuals (and all individuals included in this study) had previously been sequenced for 835 bp of mtDNA and classified into one of six regional haplotypes: IND1 (Indian); CHIE (Eastern China); CHIW1, CHIW2, and CHIW3 (all from Western China); and CHIS (Southern China) [33]. The CHIW1 sample, individual Sch00R1684, had also been used to generate the 454 fragments. The Genbank accession numbers and geographic origins for these samples are given in Table 2.

For amplification, 2 μl of DNA extract was added to a 25 μl PCR reaction with 67 mM Tris HCl, pH 8.8, 16 mM (NH_4_)_2_SO_4_, 0.01% Tween-20, 0.05 mM each dNTP, 0.2 mM each primer, 1.7 mM MgSO_4_, and 0.025 U/μl Invitrogen Platinum Taq, (Invitrogen Corp., Carlsbad, CA). Thermocycling conditions included an initial hold at 94°C for three minutes, followed by 60 cycles of fifteen seconds at 94°C, twenty seconds at 62°C, and twenty seconds at 72°C, and a final hold at 72°C for five minutes. The amplicons were digested with ExoI (0.25 U/μl PCR product) at 37°C for 90 minutes to remove any remaining primer, heated to 80°C for 20 minutes and filtered with Montage PCRμ96 plates (Millipore, Billerica, MA). They were then submitted to UC Davis Division of Biological Sciences, where they were sequenced in both the forward and reverse directions on an ABI 3730 sequencer using the Big Dye Terminator Cycle Sequencing version 3.1 kit (Applied Biosystems, Foster City, CA). The sequenced products were edited and aligned using the program Sequencher 4.7 for Macintosh. Consensus sequences were generated from the aligned products.

### Screening for Informative SNPs

Forty-eight SNP loci were chosen from pyrofragments with a Phred score of greater than 20 and at least one overlapping fragment. These SNPs included at least one arm on each chromosome (except Y). All DNA extraction, primer design, sequencing and analysis methods are as described above. Primers were designed to amplify 400 bp of flanking sequence around the SNP. The primer information for each locus is listed in Additional File 1: "Primer Information for Rhesus SNP Resequencing". DNA samples from four rhesus macaques (listed in Table 2) were extracted and combined to produce a single "pooled" sample.

For amplification, 8 μl of DNA (100 ng, 25 ng per each sample) was added to a 25 μl PCR reaction with 1× Platinum^® ^PCR Buffer, 1 U Platinum^® ^Taq DNA Polymerase, 0.1 mM each dNTP, 2.0 mM MgSO_4_, 0.2 μM each primer. Thermocycling conditions included an initial hold at 94°C for two minutes, followed by 40 cycles of fifteen seconds at 94°C, thirty seconds at 59°C, and forty seconds at 72°C, and a final hold at 72°C for five minutes. The amplicons were digested with ExoI (0.5 U/μl PCR product) and SAP (0.05 U/μl of PCR product) at 37°C for 30 minutes to remove any remaining primer and dNTPs and heated to 80°C for 15 minutes. They were then submitted to the University of Illinois Urbana-Champagne High-Throughput Sequencing Unit of the Keck Center, where they were sequenced in both the forward and reverse directions on an ABI 3730XL sequencer, using Big Dye Terminator Cycle Sequencing version 3.1 (Applied Biosystems, Foster City, CA). The sequenced products were edited and aligned using the program Sequencher 4.7 for Windows. Consensus sequences were generated from the aligned products. One 800-bp DNA sequence was produced using this pooled sample, for each of the 48 chosen SNPs. To more accurately assess the distribution of polymorphism, all four rhesus macaques were also individually sequenced at three of these SNP loci, once they were confirmed to be polymorphic.

### Detecting Geographic Variation

In a third test of our methodology, primers were made to genotype an additional 96 candidate SNPs. These 96 SNPs were chosen from the 1,559 SNPs theoretically identified through pyrofragment comparison, with a minimum PHRED score of 20 and at least one overlapping fragment. They were amplified in a sample of 95 rhesus macaques from both India and China (one individual was duplicated, for a total of 96 DNA samples), representing a regionally diverse and geographically representative sample of animals from each country. Information on these animals can be found in Table 3. Genotyping was conducted at the University of California, Davis Genome Center DNA Technologies Core using the Illumina GoldenGate Assay system (Illumina Inc., San Diego, CA). Locale information on these SNP markers is shown in Table 4. Complete information on the SNP loci used for detection of geographic variation can be found in Additional File 2: "All Validated SNPs".

The 96 animals submitted for SNP genotyping were also genotyped at 23 autosomal STR loci. The PCR reactions used 0.5–1.25 μl of DNA extract in each 12.5 μl PCR reaction [67 mM Tris HCl (pH 8.8), 16 mM(NH_4_)_2_SO_4_, 0.01% Tween-20, 0.05 mM each dNTP, 0.2 μM each primer, 1.7 mM MgSo_4_, and 0.025 units/ml Invitrogen Platinum Taq (Invitrogen Corp., Carlsbad, CA)]. Each STR primer pair was optimized for annealing temperature and extension time: 94°C for 3 min., then 60 cycles of 94°C for 20 sec., 54–62°C (-0.1°C/cycle), 72°C for 45–90 sec., and a final extension at 72°C for 5–60 sec. All samples were analyzed on an ABI 310 sequencer using the Liz 500 size standard, and the ABI GeneScan software to assign genotypes. Some of the STR genotypes included in this study were also included in [[28] and [29]]. Heterozygosity, allele frequencies, and linkage disequalibrium was calculated for the SNP data set using Genepop. Similar values for the STR data were calculated using GenePop [63]. Principle component analyses (PCAs) on both data sets were performed using the adegenet 1.1 [66] package for R.

## Authors' contributions

Experiments conceived and designed by RSM, SK, and DGS. JAS and RYT performed the experiments. RSM, JAS, RYT and VSM analyzed the data. RSM and DGS contributed reagents, materials and analysis tools. JAS, RSM, SK and DGS wrote the paper.

## Supplementary Material

Additional file 1 The file "Additional_File1.doc" has been uploaded. This file is a table in Microsoft Word format. The data is titled The file is titled: "Primer Information for Rhesus SNP Resequencing" and includes the name of the pyrofragment in which the SNP was located, the chromosome and nucleotide position, the direction of the change, the number of overlapping pyrofragments and the Phred score of the SNP. Click here for file

Additional file 2 The file "Additional_File2.doc" has been uploaded. This file is a table in Microsoft Word format. The data is titled: "All validated SNPs", and includes the chromosome, nucleotide position, the name of the 454 fragment in which the SNP was originally discovered, the polymorphism, the observed heterozygosity in the sample of Chinese animals, observed heterozygosity in the sample of Indian animals, the gene or feature in which the SNP is located (if any), the nearest genes or features at both the 5' and 3' sides (with a maximum distance of 1.5 Mb). Click here for file
