# Supplementary material for: Pyrosequencing as a method for SNP identification in the rhesus macaque (Macaca mulatta)
Source: BMC Genomics. 2008 May 29;9:256. doi: 10.1186/1471-2164-9-256 (PMC2443142; doi:10.1186/1471-2164-9-256)
Supplement: Additional file 1 — The file "Additional_File1.doc" has been uploaded. This file is a table in Microsoft Word format. The data is titled The file is titled: "Primer Information for Rhesus SNP Resequencing" and includes the name of the pyrofragment in which the SNP was located, the chromosome and nucleotide position, the direction of the change, the number of overlapping pyrofragments and the Phred score of the SNP. [file 1471-2164-9-256-S1.doc]

| **Pyrofragment ID** | **Chromosome** | **Position** | **Change** | **Overlapping Fragments** | **PHRED** |
| --- | --- | --- | --- | --- | --- |
| D8YOWMI02HZBRA | 1 | 175452 | T->C | 2 | 26 |
|  | *Unsuccessful Amplification* | | | | |
|  |  | | | | |
| D8YOWMI01E0VHD | 1 | 217935620 | G->C | 1 | 26 |
| Forward Primer: | 5’CCTCCCTGGTGTGTAGGAATAA3’ | | | | |
| Reverse Primer: | 5’CACTGTAGTCAAGGGAACATGG3’ | | | | |
| D8YOWMI01DTGIR | 2 | 1925239 | C->A | 2 | 27 |
| Forward Primer: | 5’TGGGACAGGGTTATTGAAGATT3’ | | | | |
| Reverse Primer: | 5’CATATGCCAGTTCCTCTTAGGG3’ | | | | |
| D8YOWMI02HC8IL | 2 | 160903336 | G->T | 2 | 27 |
|  | *Unsuccessful Amplification* | | | | |
|  |  | | | | |
| D8YOWMI02GSRQO | 3 | 7026913 | C->A | 1 | 26 |
| Forward Primer: | 5’TACTCAGCCCCATTCTTCCTTA3’ | | | | |
| Reverse Primer: | 5’GGCTGAAGATTCACCAGTGATA3’ | | | | |
| D8YOWMI02GNQT9 | 3 | 183283945 | A->C | 1 | 31 |
| Forward Primer: | 5’TGGTAGAAATGGCAGGATATCAA3’ | | | | |
| Reverse Primer: | 5’AGTAGGAATGGGACAGCTTGAA3’ | | | | |
| D8YOWMI02HP0CJ | 4 | 8097710 | A->G | 2 | 29 |
| Forward Primer: | 5’AATCCTGTGGTCTGTCATGTTG3’ | | | | |
| Reverse Primer: | 5’TCCTTACTGAGGACAATGCAAA3’ | | | | |
| D8YOWMI01BGHP9 | 4 | 152126904 | A->G | 2 | 29 |
| Forward Primer: | 5’GGAGCCATTAACACATCCATTT3’ | | | | |
| Reverse Primer: | 5’TGTGGGTCAAATAGCTTCTGTG3’ | | | | |
| D8YOWMI01DOPOW | 5 | 23874066 | G->C | 1 | 27 |
| Forward Primer: | 5’GAGAAACAAGCTCTCAAAAGCA3’ | | | | |
| Reverse Primer: | 5’TTTTTGATTTGGTGTCTATCCAA3’ | | | | |
| D8YOWMI01BHQA0 | 5 | 172356470 | G->T | 1 | 27 |
| Forward Primer: | 5’TCCCATTGACTTATTTTCTGAGG3’ | | | | |
| Reverse Primer: | 5’TGTAAGTGGAGGAAATGCATCA3’ | | | | |
| D8YOWMI02IWMJX | 6 | 7769739 | A->C | 1 | 26 |
| Forward Primer: | 5’AACAGAACATTTCTTCCCTTTCA3’ | | | | |
| Reverse Primer: | 5’TGAACCACTAAAAATTCCTACCC3’ | | | | |
| D8YOWMI01BSRVQ | 6 | 31257382 | G->C | 1 | 26 |
| Forward Primer: | 5’CATTTCTCCCTGGAGTTCAGTT3’ | | | | |
| Reverse Primer: | 5’CGAGACCCTAATTCTGATGGAC3’ | | | | |
| D8YOWMI01AOPOT | 6 | 40308517 | G->A | 2 | 27 |
| Forward Primer: | 5’GCATGCCTCCACACTTATTTCT3’ | | | | |
| Reverse Primer: | 5’TTTCTCTGTGATGGCTATCGTG3’ | | | | |
| D8YOWMI02FG8XU | 6 | 141995808 | A->C | 1 | 27 |
| Forward Primer: | 5’CCAAATTGGACATTTCTTGACC3’ | | | | |
| Reverse Primer: | 5’AGATGAACTCCAAAAACCAGGA3’ | | | | |
| D8YOWMI01CG0QE | 6 | 151694674 | C->A | 1 | 27 |
| Forward Primer: | 5’GTGGCTCTACCCACTTCCTCTA3’ | | | | |
| Reverse Primer: | 5’GGTGCACTAGAGGACAACAATG3’ | | | | |

| **Pyrofragment ID** | **Chromosome** | **Position** | **Change** | **Overlapping Fragments** | **PHRED** |
| --- | --- | --- | --- | --- | --- |
| D8YOWMI02J5FHH | 7 | 13009898 | T->G | 1 | 31 |
| Forward Primer: | 5’TTGAAGATCTCACCTCCTTCG3’ | | | | |
| Reverse Primer: | 5’TCCCTCTTTTCTCAAACCTCA3’ | | | | |
| D8YOWMI01A6K13 | 7 | 160359546 | T->G | 1 | 26 |
| Forward Primer: | 5’ACCTTCTTGCAGTTCCAGAGAC3’ | | | | |
| Reverse Primer: | 5’ATGTCTTCAAGGGAGCAAGAAA3’ | | | | |
| D8YOWMI02JZ4L3 | 8 | 5736461 | G->C | 1 | 26 |
| Forward Primer: | 5’TTGGGAGGAGGGAATTAAGTTT3’ | | | | |
| Reverse Primer: | 5’ACTGAGGTTTGTTTTGCCAGTT3’ | | | | |
| D8YOWMI02FRKIK | 8 | 93167230 | G->A | 2 | 27 |
| Forward Primer: | 5’CAGATGCAGTACTCAGCCTCAT3’ | | | | |
| Reverse Primer: | 5’GGAATTTGTGGGAACCATTTAT3’ | | | | |
| D8YOWMI02GWSTP | 9 | 3182742 | C->G | 1 | 31 |
| Forward Primer: | 5’AACAGCCTGGTCAGTAGATGGT3’ | | | | |
| Reverse Primer: | 5’TCCAGTTGGAGGTAGATTTGGT3’ | | | | |
| D8YOWMI02IVOYO | 9 | 120981824 | A->C | 1 | 26 |
| Forward Primer: | 5’ CGACTCCATTTCCCATGTTC 3’ | | | | |
| Reverse Primer: | 5’ CTATGCAGCCCCATTCCTAA 3’ | | | | |
| D8YOWMI02FZMH9 | 10 | 937132 | G->T | 1 | 27 |
| Forward Primer: | 5’ CACAAAACTTTGGGGGTCTTG 3’ | | | | |
| Reverse Primer: | 5’ AGGCTCTGGGAAGAAAGTGTC 3’ | | | | |
| D8YOWMI01B6Z1V | 10 | 69095838 | T->C | 2 | 31 |
| Forward Primer: | 5’ GAAGAGAGTGGCACACCCATA 3’ | | | | |
| Reverse Primer: | 5’ CCCCAGTAATGTCTGGGATCT 3’ | | | | |
| D8YOWMI02HKHPD | 11 | 4331038 | T->C | 2 | 26 |
| Forward Primer: | 5’ TAATGTTGCCACAAATTGATCC 3’ | | | | |
| Reverse Primer: | 5’ CTCAGGGCAGCTCTCTCTCTCT 3’ | | | | |
| D8YOWMI01AQR0L | 11 | 98846449 | T->C | 3 | 32 |
| Forward Primer: | 5’ CGCTTTTGTGAAGTTCTGTTGT 3’ | | | | |
| Reverse Primer: | 5’ CCTGAAGAAATGAAGGTGGAAG 3’ | | | | |
| D8YOWMI01EQOOR | 12 | 2820172 | C->A | 1 | 25 |
| Forward Primer: | 5’ AAAAACTGTCCTCCCCACAATA 3’ | | | | |
| Reverse Primer: | 5’ TCCACTGGATGTATATGGAAACT 3’ | | | | |
| D8YOWMI02HAIYA | 12 | 104866834 | C->T | 1 | 27 |
|  | *Unsuccessful Amplification* | | | | |
|  |  | | | | |
| D8YOWMI01ETU8L | 13 | 6457948 | G->T | 2 | 33 |
| Forward Primer: | 5’ TTCCTTTCAACTTTGTTTCAAAT 3’ | | | | |
| Reverse Primer: | 5’ TTGATCAAAGTTTCAGAGGTTTG 3’ | | | | |
| D8YOWMI02FR1F9 | 13 | 131806911 | C->G | 1 | 33 |
| Forward Primer: | 5’ GAGCATACAGGTCCAATTCCA 3’ | | | | |
| Reverse Primer: | 5’ TGGCTTTCCAGAAAGGAAGT 3’ | | | | |
| D8YOWMI02G5IJG | 14 | 27578133 | A->T | 1 | 30 |
|  | *Unsuccessful Amplification* | | | | |
|  |  | | | | |

| **Pyrofragment ID** | **Chromosome** | **Position** | **Change** | **Overlapping Fragments** | **PHRED** |
| --- | --- | --- | --- | --- | --- |
| D8YOWMI02I6VU7 | 14 | 132507313 | A->T | 1 | 27 |
|  | *Unsuccessful Amplification* | | | | |
|  |  | | | | |
| D8YOWMI02F2CCM | 15 | 4214368 | G->C | 1 | 28 |
| Forward Primer: | 5’ GTTTTCCCTTCCCTACTCCACT 3’ | | | | |
| Reverse Primer: | 5’ CTTTGTGGGGGAGCTAGAAAC 3’ | | | | |
| D8YOWMI01E4TU3 | 15 | 102228356 | G->C | 2 | 31 |
| Forward Primer: | 5’ GGAGTCAAACGCAGTATGTGAA 3’ | | | | |
| Reverse Primer: | 5’ TATATTCCGCAGACAAATGCAG 3’ | | | | |
| D8YOWMI02F8F0W | 16 | 41616577 | C->A | 2 | 27 |
| Forward Primer: | 5’ CATCAGAAACTGATTGCCATGT 3’ | | | | |
| Reverse Primer: | 5’ CTGGCTGAAATAACACAAGCTG 3’ | | | | |
| D8YOWMI01BSPN1 | 16 | 73114616 | G->A | 3 | 27 |
| Forward Primer: | 5’ CTAAACACATGGGCGTACTTGG 3’ | | | | |
| Reverse Primer: | 5’ GGAAAGCGTGGAAATACTCAAG 3’ | | | | |
| D8YOWMI01BBO36 | 17 | 33408638 | A->C | 1 | 27 |
| Forward Primer: | 5’ TGCTTTGAGTTAAGCAAACTGC 3’ | | | | |
| Reverse Primer: | 5’ TCCAATGTTTTCTTTTTGTTTTTG 3’ | | | | |
| D8YOWMI02JABAC | 17 | 86832829 | T->C | 2 | 26 |
| Forward Primer: | 5’ GCAATTTTTCATGGACCTAATCA 3’ | | | | |
| Reverse Primer: | 5’ AATACCGGACCTGCAAACTAAA 3’ | | | | |
| D8YOWMI02IZYSI | 18 | 7762691 | C->G | 1 | 26 |
| Forward Primer: | 5’ AGATGTGCAGGAAAAAGGAAAC 3’ | | | | |
| Reverse Primer: | 5’ GGGGAGTGTCTTCAGGATTTTA 3’ | | | | |
| D8YOWMI01EI6PE | 18 | 55288574 | T->A | 1 | 30 |
| Forward Primer: | 5’3’ | | | | |
| Reverse Primer: | 5’3’ | | | | |
| D8YOWMI01ADGFV | 18 | 68548987 | G->A | 2 | 27 |
| Forward Primer: | 5’ GAGGCAAGAAATACCCTTCGT 3’ | | | | |
| Reverse Primer: | 5’ GTCAAGTCTACCACCGTCCTG 3’ | | | | |
| D8YOWMI01ELUBW | 19 | 5832169 | G->T | 1 | 27 |
| Forward Primer: | 5’ TCTCTGGGAATGTGTACAGTGG 3’ | | | | |
| Reverse Primer: | 5’ ACACACACTGCTCATCAGGAAC 3’ | | | | |
| D8YOWMI01A5JSC | 19 | 38104151 | C->G | 1 | 27 |
| Forward Primer: | 5’ CTTATTGGCGGTGTCTGTGAT 3’ | | | | |
| Reverse Primer: | 5’ TCCAGGGTAAAGTGTGAAATCC 3’ | | | | |
| D8YOWMI02FS2LX | 19 | 60457694 | G->T | 1 | 31 |
| Forward Primer: | 5’ TGCATTGCTTTAAATGTTTCAGA 3’ | | | | |
| Reverse Primer: | 5’ CCGGCTCCTACCAATAACTATG 3’ | | | | |
| D8YOWMI02H9SKX | 20 | 16845675 | A->T | 2 | 26 |
| Forward Primer: | 5’ AACCGAACACCTTTCTCAGTGT 3’ | | | | |
| Reverse Primer: | 5’ CAATGGCATTATGCAAGGGTAT 3’ | | | | |
| D8YOWMI01DXAXQ | 20 | 83498115 | G->T | 1 | 32 |
| Forward Primer: | 5’ CCACTGCCATGCTGTCTG 3’ | | | | |
| Reverse Primer: | 5’ TAGGTGGCTTGCATTAAATCCT 3’ | | | | |

| **Pyrofragment ID** | **Chromosome** | **Position** | **Change** | **Overlapping Fragments** | **PHRED** |
| --- | --- | --- | --- | --- | --- |
| D8YOWMI01A5TTU | X | 2357083 | G->T | 1 | 30 |
| Forward Primer: | 5’ TATGGCCTGAAGGTATCAGACA 3’ | | | | |
| Reverse Primer: | 5’ GACCAGGATAAAGTGAATGTTGG 3’ | | | | |
| D8YOWMI01B3P65 | X | 13953777 | C->T | 2 | 27 |
| Forward Primer: | 5’3’ | | | | |
| Reverse Primer: | 5’3’ | | | | |
| D8YOWMI02GK6I6 | X | 22094791 | G->T | 1 | 26 |
| Forward Primer: | 5’ GGTCACAGTTCTTTGTGTCTGG3’ | | | | |
| Reverse Primer: | 5’ TATGAGAGAAGTCCCCGAGAGA3’ | | | | |
| D8YOWMI01C7Z8G | X | 90299244 | C->G | 1 | 26 |
| Forward Primer: | 5’ GCTGTGCTTTTCTGGTCTTCTAA3’ | | | | |
| Reverse Primer: | 5’ AGCTCTTACATGCAGAGCCTTT3’ | | | | |
