# Supplementary material for: Pyrosequencing as a method for SNP identification in the rhesus macaque (Macaca mulatta)
Source: BMC Genomics. 2008 May 29;9:256. doi: 10.1186/1471-2164-9-256 (PMC2443142; doi:10.1186/1471-2164-9-256)
Supplement: Additional file 2 — The file "Additional_File2.doc" has been uploaded. This file is a table in Microsoft Word format. The data is titled: "All validated SNPs", and includes the chromosome, nucleotide position, the name of the 454 fragment in which the SNP was originally discovered, the polymorphism, the observed heterozygosity in the sample of Chinese animals, observed heterozygosity in the sample of Indian animals, the gene or feature in which the SNP is located (if any), the nearest genes or features at both the 5' and 3' sides (with a maximum distance of 1.5 Mb). [file 1471-2164-9-256-S2.doc]

| **Chrom** | **Position** | **454**  **Fragment** | **Change** | **China**  **H_O_** | **India**  **H_O_** | **Gene or Feature** |
| --- | --- | --- | --- | --- | --- | --- |
|  |  |  |  |  |  | **5’ Gene or Feature** |
|  |  |  |  |  |  | **3’ Gene or Feature** |
| 1 | 41591807 | D8YOWMI01CKGJZ | T->C | 0.35 | 0.54 |  |
|  |  |  |  |  |  | 665224 bp: similar to POU domain, class 3, transcription factor 1 |
|  |  |  |  |  |  | 110708 bp: similar to Ras-related GTP binding protein |
| 1 | 83688735 | D8YOWMI02IW5CF | C->T | 0.28 | 0.05 |  |
|  |  |  |  |  |  |  |
|  |  |  |  |  |  |  |
| 1 | 137720099 | D8YOWMI02F9MU6 | C->A | 0.49 | 0.46 |  |
|  |  |  |  |  |  | 50713 bp: similar to absent in melanoma 2 |
|  |  |  |  |  |  | 55521 bp: similar to immunoglobulin superfamily, member 4B |
| 2 | 81599877 | D8YOWMI02HYHJG | C->G | 0.00 | 0.32 |  |
|  |  |  |  |  |  |  |
|  |  |  |  |  |  |  |
| 2 | 102042961 | D8YOWMI02FSUU7 | A->C | 0.23 | 0.30 |  |
|  |  |  |  |  |  | 101591 bp: melanoma antigen family F, 1 |
|  |  |  |  |  |  | 25998 bp: similar to ephrin receptor EphB3 precursor |
| 2 | 107124770 | D8YOWMI01BCK71 | T->A | 0.23 | 0.38 |  |
|  |  |  |  |  |  | 6272 bp: ubiquitin specific protease 13 (isopeptidase T-3) |
|  |  |  |  |  |  |  |
| 2 | 108271889 | D8YOWMI02HSRZI | T->G | 0.09 | 0.00 |  |
|  |  |  |  |  |  | 278651 bp: similar to calcium-activated potassium channel beta 2 subunit |
|  |  |  |  |  |  |  |
| 2 | 173429669 | D8YOWMI02H9ZDI | C->T | 0.00 | 0.00 |  |
|  |  |  |  |  |  | 112568 bp: similar to glutamate decarboxylase-like 1 |
|  |  |  |  |  |  | 503278 bp: similar to Thyroid hormone receptor-associated protein 3 |
| 3 | 2786166 | D8YOWMI02IQPMR | T->C | 0.00 | 0.00 |  |
|  |  |  |  |  |  |  |
|  |  |  |  |  |  | 123296 bp: nucleolar protein NOP52 |
| 3 | 5541516 | D8YOWMI01CIYVH | T->C | 0.49 | 0.49 |  |
|  |  |  |  |  |  | 61775 bp: similar to beta-site APP-cleaving enzyme 2 isoform C |
|  |  |  |  |  |  |  |
| 3 | 92289501 | D8YOWMI02GYWN8 | G->T | 0.33 | 0.11 |  |
|  |  |  |  |  |  | 57161 bp: similar to BMP-binding endothelial regulator precursor |
|  |  |  |  |  |  |  |
| 3 | 109489675 | D8YOWMI01A3RKI | C->A | 0.00 | 0.00 | similar to ankyrin repeat and MYND domain containing 2 |
|  |  |  |  |  |  |  |
|  |  |  |  |  |  |  |
| 3 | 113721716 | D8YOWMI02HBRPH | T->A | 0.35 | 0.59 |  |
|  |  |  |  |  |  |  |
|  |  |  |  |  |  | 41119 bp: similar to 40S ribosomal protein S4, X isoform 1 |
| 4 | 8667085 | D8YOWMI01D7KJS | C->T | 0.09 | 0.03 |  |
|  |  |  |  |  |  |  |
|  |  |  |  |  |  |  |
| 4 | 70905133 | D8YOWMI01B5BA3 | G->C | 0.00 | 0.49 |  |
|  |  |  |  |  |  | 832604 bp: similar to CD109 isoform 1 |
|  |  |  |  |  |  | 315996 bp similar to Collagen alpha-1(XII) chain precursor |
| 4 | 97060095 | D8YOWMI01CYV2Z | A->C | 0.26 | 0.05 |  |
|  |  |  |  |  |  | 668755 bp: similar to single-minded homolog 1 |
|  |  |  |  |  |  | 284032 bp: similar to glutamate receptor 6 isoform 1 precursor |
| 4 | 100849065 | D8YOWMI02HW5RE | C->A | 0.00 | 0.00 |  |
|  |  |  |  |  |  | 1306928 bp: similar to 60S ribosomal protein L7a |
|  |  |  |  |  |  | 128306 bp: similar to lin-28 homolog B |
| 4 | 151067488 | D8YOWMI01BM8Y1 | G->A | 0.05 | 0.00 |  |
|  |  |  |  |  |  | 977543 bp: similar to ribosomal protein L30 |
|  |  |  |  |  |  | 521053 bp: similar to ret finger protein-like 4 |
| 5 | 130251597 | D8YOWMI01DMHC1 | T->C | 0.00 | 0.00 |  |
|  |  |  |  |  |  | 277983 bp: similar to protocadherin 18 precursor |
|  |  |  |  |  |  | 351178 bp: solute carrier family 7 |
| 5 | 130308604 | D8YOWMI01A4SPO | G->A | 0.07 | 0.00 |  |
|  |  |  |  |  |  | 335041 bp: similar to protocadherin 18 precursor |
|  |  |  |  |  |  | 294106 bp: solute carrier family 7 |
| 5 | 134028290 | D8YOWMI02FNWE0 | T->C | 0.54 | 0.03 |  |
|  |  |  |  |  |  | 336287 bp: zinc finger protein 330 isoform 2 |
|  |  |  |  |  |  | 146851 bp: similar to interleukin 15 preproprotein isoform 2 |
| 5 | 149294565 | D8YOWMI02GZALZ | C->T | 0.00 | 0.00 |  |
|  |  |  |  |  |  | 101736 bp: hypothetical protein |
|  |  |  |  |  |  | 8996 bp: glycine receptor, beta isoform 1 |
| 5 | 150489328 | D8YOWMI02IY629 | T->C | 0.35 | 0.24 |  |
|  |  |  |  |  |  | 26437 bp: hypothetical protein isoform 2 |
|  |  |  |  |  |  | 219301 bp: similar to leucine-rich repeat-containing G protein-coupled receptor |
| 5 | 154752218 | D8YOWMI01A5VX2 | C->G | 0.14 | 0.03 |  |
|  |  |  |  |  |  | 438702 bp: follistatin-like 5 |
|  |  |  |  |  |  | 614114 bp: similar to CG10341-PA |
| 6 | 48494032 | D8YOWMI01BK976 | G->A | 0.05 | 0.19 |  |
|  |  |  |  |  |  | 282141 bp: hypothetical protein |
|  |  |  |  |  |  | 335920 bp: similar to 40S ribosomal protein S10 |
| 6 | 50091915 | D8YOWMI02G6KI6 | C->A | 0.40 | 0.30 |  |
|  |  |  |  |  |  | 374901 bp: similar to ubiquitin-conjugating enzyme E2N |
|  |  |  |  |  |  | 331622 bp: similar to integrin, alpha 1 precursor |
| 6 | 69342401 | D8YOWMI01ADMRV | T->G | 0.09 | 0.19 |  |
|  |  |  |  |  |  | 1733 bp: hypothetical protein isoform 2 |
|  |  |  |  |  |  | 241657 bp: similar to Nonhistone chromosomal protein HMG-17 |
| 6 | 71262852 | D8YOWMI01EEJS0 | T->G | 0.47 | 0.38 |  |
|  |  |  |  |  |  | 62320 bp: similar to 60S ribosomal protein L27a |
|  |  |  |  |  |  | 93464 bp: similar to Ankyrin repeat domain-containing protein 11 |
| 6 | 162595742 | D8YOWMI01CTS8T | G->A | 0.26 | 0.35 |  |
|  |  |  |  |  |  | 2734465 bp: similar to methionine adenosyltransferase II, beta isoform |
|  |  |  |  |  |  | 1052908 bp similar to odd Oz/ten-m homolog 2 isoform 4 |
| 7 | 66188899 | D8YOWMI02H6Q3M | G->A | 0.26 | 0.51 | similar to ATP/GTP binding protein 1 |
|  |  |  |  |  |  |  |
|  |  |  |  |  |  |  |
| 7 | 73733494 | D8YOWMI02IY81A | A->G | 0.44 | 0.32 |  |
|  |  |  |  |  |  | 1220130 bp: hypothetical protein isoform 1 |
|  |  |  |  |  |  | 423491 bp: similar to multiple C2-domains with two trans-membrane regions |
| 7 | 88858093 | D8YOWMI01A8Y8T | A->G | 0.29 | 0.47 |  |
|  |  |  |  |  |  | 893170 bp: similar to amisyn |
|  |  |  |  |  |  | 472572 bp: similar to neuro-oncological ventral antigen 1 isoform 1 |
| 7 | 90512305 | D8YOWMI01AK1JJ | C->T | 0.51 | 0.36 |  |
|  |  |  |  |  |  | 185426 bp: similar to high-mobility group box 2 |
|  |  |  |  |  |  |  |
| 7 | 96142386 | D8YOWMI02JB3L7 | A->C | 0.00 | 0.00 | neuronal PAS domain protein 3 |
|  |  |  |  |  |  |  |
|  |  |  |  |  |  |  |
| 8 | 15924443 | D8YOWMI02JPNQ1 | G->C | 0.00 | 0.00 |  |
|  |  |  |  |  |  | 253563 bp: similar to protein phosphatase 1A isoform 1 |
|  |  |  |  |  |  | 50945 bp: macrophage scavenger receptor 1 |
| 8 | 17121998 | D8YOWMI01EUPYP | A->G | 0.47 | 0.41 | similar to hepatocellular carcinoma related protein 1 |
|  |  |  |  |  |  |  |
|  |  |  |  |  |  |  |
| 8 | 36527694 | D8YOWMI02JANH7 | T->C | 0.00 | 0.22 |  |
|  |  |  |  |  |  | 367662 bp: netrin receptor Unc5h4 |
|  |  |  |  |  |  | 670468 bp: similar to large conductance calcium-activated potassium channels |
| 8 | 40839858 | D8YOWMI01DPR0X | C->T | 0.16 | 0.49 |  |
|  |  |  |  |  |  | 137411 bp: similar to Protein C8orf4 |
|  |  |  |  |  |  | 240139 bp: similar to zinc finger, matrin type 4 |
| 8 | 118627401 | D8YOWMI02IYG8H | C->T | 0.00 | 0.00 |  |
|  |  |  |  |  |  | 508721 bp: zinc finger transcription factor TRPS1 |
|  |  |  |  |  |  | 532310 bp: similar to eukaryotic translation initiation factor 3 |
| 9 | 1893975 | D8YOWMI02JQRA8 | T->C | 0.30 | 0.06 |  |
|  |  |  |  |  |  | 118885 bp: hypothetical protein |
|  |  |  |  |  |  |  |
| 9 | 48807739 | D8YOWMI02HME0K | A->T | 0.47 | 0.11 |  |
|  |  |  |  |  |  | 217933 bp: similar to protein tyrosine phosphatase, non-receptor type |
|  |  |  |  |  |  | 88833 bp: growth differentiation factor 10 |
| 9 | 54875394 | D8YOWMI02IMO25 | C->T | 0.47 | 0.41 |  |
|  |  |  |  |  |  |  |
|  |  |  |  |  |  |  |
| 9 | 82319123 | D8YOWMI01CFSTX | A->T | 0.16 | 0.49 |  |
|  |  |  |  |  |  | 1333861 bp: similar to ZW10 interactor |
|  |  |  |  |  |  | 327879 bp: similar to protocadherin 15 precursor |
| 10 | 28476219 | D8YOWMI02H5GST | G->T | 0.00 | 0.16 |  |
|  |  |  |  |  |  |  |
|  |  |  |  |  |  | 13783 bp: hypothetical protein LOC140894 isoform 3 |
| 10 | 39764434 | D8YOWMI02J18DD | T->C | 0.00 | 0.19 |  |
|  |  |  |  |  |  | 337359 bp: similar to chromosome 20 open reading frame 42 isoform 3 |
|  |  |  |  |  |  | 312676 bp: bone morphogenetic protein 2 |
| 10 | 43811179 | D8YOWMI02ILVBH | G->T | 0.40 | 0.54 | hypothetical protein |
|  |  |  |  |  |  |  |
|  |  |  |  |  |  |  |
| 10 | 57117826 | D8YOWMI02F0DVR | G->C | 0.81 | 0.54 |  |
|  |  |  |  |  |  |  |
|  |  |  |  |  |  |  |
| 11 | 36838935 | D8YOWMI01BPZY1 | T->C | 0.00 | 0.00 | similar to solute carrier family 2 |
|  |  |  |  |  |  |  |
|  |  |  |  |  |  |  |
| 11 | 59714175 | D8YOWMI01B2MKG | T->A | 0.00 | 0.00 |  |
|  |  |  |  |  |  | 115900 bp: ubiquitin specific peptidase 15 isoform 1 |
|  |  |  |  |  |  | 77526 bp: hypothetical protein |
| 11 | 63944188 | D8YOWMI01AE8L5 | C->T | 0.33 | 0.49 |  |
|  |  |  |  |  |  | 264041 bp: glutamate receptor interacting protein 1 isoform 4 |
|  |  |  |  |  |  | 112042 bp: hypothetical protein |
| 11 | 64500686 | D8YOWMI02FNXIN | G->C | 0.21 | 0.30 |  |
|  |  |  |  |  |  | 189980 bp: similar to TIP120 protein |
|  |  |  |  |  |  | 138585 bp: dual-specificity tyrosine-(Y)-phosphorylation regulated kinase |
| 11 | 64700872 | D8YOWMI01BKBY2 | C->A | 0.05 | 0.32 |  |
|  |  |  |  |  |  | 51888 bp: dual-specificity tyrosine-(Y)-phosphorylation regulated kinase |
|  |  |  |  |  |  | 443017 bp: interferon-gamma |
| 11 | 127577385 | D8YOWMI01A2KPT | A->G | 0.00 | 0.38 |  |
|  |  |  |  |  |  | 610506 bp: similar to CG14446-PA |
|  |  |  |  |  |  |  |
| 12 | 3251136 | D8YOWMI01ARFTU | T->C | 0.26 | 0.03 |  |
|  |  |  |  |  |  |  |
|  |  |  |  |  |  |  |
| 12 | 32363345 | D8YOWMI01D7OOM | T->A | 0.00 | 0.22 | similar to longevity assurance homolog 6 isoform 2 |
|  |  |  |  |  |  |  |
|  |  |  |  |  |  |  |
| 12 | 38358965 | D8YOWMI01E3M7Q | A->G | 0.14 | 0.30 |  |
|  |  |  |  |  |  | 160844 bp: similar to Wiskott-Aldrich syndrome protein-interacting protein |
|  |  |  |  |  |  | 15015 bp: similar to cholinergic receptor, nicotinic, alpha 1 |
| 13 | 39437602 | D8YOWMI02JONIL | T->A | 0.30 | 0.03 | similar to mitogen-activated protein kinase |
|  |  |  |  |  |  |  |
|  |  |  |  |  |  |  |
| 13 | 43055931 | D8YOWMI02FUT4G | G->C | 0.00 | 0.00 |  |
|  |  |  |  |  |  | 109902 bp: similar to cysteine and histidine-rich domain (CHORD)-containing, zinc-binding protein 1 |
|  |  |  |  |  |  |  |
| 13 | 43184712 | D8YOWMI01DBUQX | C->T | 0.09 | 0.14 |  |
|  |  |  |  |  |  | 238695 bp: similar to cysteine and histidine-rich domain (CHORD)-containing, zinc-binding protein 1 |
|  |  |  |  |  |  |  |
| 13 | 64444405 | D8YOWMI01A1PLP | A->C | 0.23 | 0.30 |  |
|  |  |  |  |  |  | 86138 bp: similar to pellino protein |
|  |  |  |  |  |  | 219407 bp: similar to Galectin-5 (RL-18) |
| 13 | 77238559 | D8YOWMI02H6KYZ | C->T | 0.00 | 0.00 |  |
|  |  |  |  |  |  | 156125 bp: similar to Leucine-rich repeat transmembrane neuronal protein |
|  |  |  |  |  |  | 634149 bp: leucine rich repeat transmembrane neuronal 4 isoform 2 |
| 14 | 27578189 | D8YOWMI02G5IJG | T->A | 0.47 | 0.49 |  |
|  |  |  |  |  |  | 68461 bp: CD82 molecule isoform 1 |
|  |  |  |  |  |  |  |
| 14 | 30474050 | D8YOWMI01E00NZ | T->A | 0.19 | 0.00 |  |
|  |  |  |  |  |  | 142118 bp: similar to DNA damage-binding protein 2 |
|  |  |  |  |  |  | 1382048 bp: similar to netrin-G1 ligand isoform 1 |
| 14 | 32909061 | D8YOWMI01DUPZD | T->C | 0.12 | 0.57 |  |
|  |  |  |  |  |  | 1051008 bp: similar to netrin-G1 ligand isoform 4 |
|  |  |  |  |  |  |  |
| 14 | 42631928 | D8YOWMI02HA390 | C->T | 0.00 | 0.03 |  |
|  |  |  |  |  |  | 126056 bp: similar to ATP synthase, H+ transporting, mitochondrial F0 complex |
|  |  |  |  |  |  | 819278 bp: similar to methyltransferase 5 domain containing 1 |
| 14 | 64204698 | D8YOWMI02FNMDV | A-.T | 0.00 | 0.35 |  |
|  |  |  |  |  |  | 45519 bp: similar to Olfactory receptor 5P2 |
|  |  |  |  |  |  |  |
| 14 | 118379029 | D8YOWMI02HPAC6 | A->G | 0.02 | 0.46 |  |
|  |  |  |  |  |  | 253459 bp at 5' side: poliovirus receptor-related 1 |
|  |  |  |  |  |  | 138697 bp: similar to tripartite motif protein TRIM29 isoform alpha variant |
| 15 | 27970376 | D8YOWMI01AZKLE | T->C | 0.00 | 0.19 |  |
|  |  |  |  |  |  | 608995 bp: actin-like 7B |
|  |  |  |  |  |  | 737130 bp: Kruppel-like factor 4 (gut) isoform 2 |
| 15 | 28423983 | D8YOWMI02GRXTR | A->C | 0.12 | 0.27 |  |
|  |  |  |  |  |  | 1062660 bp: actin-like 7B |
|  |  |  |  |  |  | 283470 bp: Kruppel-like factor 4 (gut) isoform 2 |
| 15 | 53664533 | D8YOWMI02GAT3X | C->A | 0.05 | 0.00 |  |
|  |  |  |  |  |  |  |
|  |  |  |  |  |  | 935104 bp: similar to DMRT-like family A1 |
| 15 | 87744487 | D8YOWMI01CKM4U | T->C | 0.28 | 0.38 |  |
|  |  |  |  |  |  | 727168 bp: similar to phosphoserine aminotransferase isoform 2 |
|  |  |  |  |  |  | 539344 bp: hypothetical protein |
| 16 | 46475753 | D8YOWMI01A1YBV | C->T | 0.26 | 0.00 |  |
|  |  |  |  |  |  | 10923 bp: similar to thyroid hormone receptor interactor 3 isoform 2 |
|  |  |  |  |  |  | 33853 bp: similar to phosphatidylinositol glycan, class W |
| 16 | 51833022 | D8YOWMI02H43G5 | C->A | 0.00 | 0.00 |  |
|  |  |  |  |  |  | 15859 bp: gastrin |
|  |  |  |  |  |  | 15377 bp: similar to junction plakoglobin |
| 16 | 64962285 | D8YOWMI02I1LB2 | G->A | 0.33 | 0.03 |  |
|  |  |  |  |  |  | 165366 bp: similar to mitogen-activated protein kinase 6 |
|  |  |  |  |  |  | 397557 bp: potassium inwardly-rectifying channel J16 isoform 4 |
| 17 | 54071435 | D8YOWMI01CZNUE | C->A | 0.09 | 0.49 |  |
|  |  |  |  |  |  | 840125 bp: KIAA1008 isoform 1 |
|  |  |  |  |  |  | 75657 bp: similar to Kruppel-like factor 12 isoform a isoform 3 |
| 17 | 56466655 | D8YOWMI01EXQZC | T->C | 0.46 | 0.03 |  |
|  |  |  |  |  |  | 286180 bp: hypothetical protein |
|  |  |  |  |  |  | 904179 bp: similar to potassium channel tetramerisation domain containing |
| 17 | 60683730 | D8YOWMI02HZL09 | T->A | 0.23 | 0.00 |  |
|  |  |  |  |  |  | 628205 bp: Nedd4 family interacting protein 2 |
|  |  |  |  |  |  | 132043 bp: similar to Sprouty homolog 2 (Spry-2) |
| 17 | 78807637 | D8YOWMI02IHGPU | C->G | 0.28 | 0.54 | similar to FERM, RhoGEF, and pleckstrin domain protein 1 |
|  |  |  |  |  |  |  |
|  |  |  |  |  |  |  |
| 18 | 34384603 | D8YOWMI01BQVQM | T->C | 0.00 | 0.38 |  |
|  |  |  |  |  |  | 1803823 bp: similar to haloacid dehalogenase-like hydrolase domain containing |
|  |  |  |  |  |  | 543800 bp: similar to phosphoinositide-3-kinase, class 3 |
| 18 | 40791079 | D8YOWMI02HNRPH | G->C | 0.51 | 0.32 |  |
|  |  |  |  |  |  |  |
|  |  |  |  |  |  | 88233 bp: Sma- and Mad-related protein 2 isoform 3 |
| 18 | 62691486 | D8YOWMI01D4UQY | T->C | 0.14 | 0.59 |  |
|  |  |  |  |  |  | 207529 bp: hypothetical protein LOC79839 |
|  |  |  |  |  |  | 164556 bp: similar to S-phase kinase-associated protein 1A isoform b |
| 18 | 70305391 | D8YOWMI02G2KR5 | C->G | 0.05 | 0.54 | similar to zinc finger protein 160 |
|  |  |  |  |  |  |  |
|  |  |  |  |  |  |  |
| 19 | 38754010 | D8YOWMI02FYHIB | C->T | 0.37 | 0.35 |  |
|  |  |  |  |  |  | 600984 bp: hypothetical protein |
|  |  |  |  |  |  | 115770 bp: similar to zinc finger protein 507 |
| 20 | 5921889 | D8YOWMI02GTAC8 | G->A | 0.09 | 0.19 |  |
|  |  |  |  |  |  | 463979 bp: similar to U1 small nuclear ribonucleoprotein C |
|  |  |  |  |  |  | 234886 bp: hypothetical protein |
| 20 | 5961411 | D8YOWMI01DSO9K | A->G | 0.33 | 0.46 |  |
|  |  |  |  |  |  |  |
|  |  |  |  |  |  |  |
| 20 | 60198088 | D8YOWMI02FOMNO | T->G | 0.23 | 0.16 | cadherin 8, type 2 isoform 6 |
|  |  |  |  |  |  |  |
|  |  |  |  |  |  |  |
| 20 | 76604066 | D8YOWMI01CE656 | C->G | 0.02 | 0.00 | similar to WW-domain oxidoreductase |
|  |  |  |  |  |  |  |
|  |  |  |  |  |  |  |
| 20 | 80884933 | D8YOWMI01EPJ9W | A->C | 0.49 | 0.59 | cadherin 13 |
|  |  |  |  |  |  |  |
|  |  |  |  |  |  |  |
| X | 35205919 | D8YOWMI01B87BD | C->A | 0.00 | 0.00 |  |
|  |  |  |  |  |  | 3436 bp: similar to Transmembrane gamma-carboxyglutamic acid protein |
|  |  |  |  |  |  |  |
| X | 35520501 | D8YOWMI01CTWTF | C->G | 0.19 | 0.17 |  |
|  |  |  |  |  |  | 32436 bp: similar to McLeod syndrome-associated, Kell blood group protein |
|  |  |  |  |  |  | 15088 bp: cytochrome b-245, beta polypeptide |
| X | 51763426 | D8YOWMI02IQYRA | A->G | 0.00 | 0.00 |  |
|  |  |  |  |  |  | 170 bp: hydroxyacyl-Coenzyme A dehydrogenase, type II isoform 1 |
|  |  |  |  |  |  | 85015 bp: similar to HECT, UBA and WWE domain containing 1 isoform 2 |
| X | 91273468 | D8YOWMI02HOSZS | G->A | 0.26 | 0.19 |  |
|  |  |  |  |  |  | 738717 bp: hypothetical protein |
|  |  |  |  |  |  | 179169 bp: similar to mitochondrial translational release factor 1-like |
